# Stratification of alopecia areata reveals involvement of CD4 T cell populations and altered faecal microbiota

**DOI:** 10.1093/cei/uxac088

**Published:** 2022-10-06

**Authors:** K A Bain, B Nichols, F Moffat, C Kerbiriou, U Z Ijaz, K Gerasimidis, I B McInnes, A Åstrand, S Holmes, S W F Milling

**Affiliations:** Institute of Infection, Immunity and Inflammation, University of Glasgow, Glasgow, UK; Human Nutrition, New Lister Building, University of Glasgow, Glasgow Royal Infirmary, Glasgow, UK; Alan Lyell Centre for Dermatology, Queen Elizabeth University Hospital, Glasgow, UK; Human Nutrition, New Lister Building, University of Glasgow, Glasgow Royal Infirmary, Glasgow, UK; Department of Infrastructure and Environment, University of Glasgow, Glasgow, UK; Human Nutrition, New Lister Building, University of Glasgow, Glasgow Royal Infirmary, Glasgow, UK; Institute of Infection, Immunity and Inflammation, University of Glasgow, Glasgow, UK; Late Phase Development, Respiratory, Immunology & Infection, BioPharmaceuticals R&D, AstraZeneca, Gothenburg, Sweden; Alan Lyell Centre for Dermatology, Queen Elizabeth University Hospital, Glasgow, UK; Institute of Infection, Immunity and Inflammation, University of Glasgow, Glasgow, UK

**Keywords:** T cells, autoimmunity, microbiome, skin

## Abstract

Alopecia areata (AA) is an immune-mediated disease that causes non-scarring hair loss. Autoreactive CD8 T cells are key pathogenic effectors in the skin, and AA has been associated both with atopy and with perturbations in intestinal homeostasis. This study aimed to investigate mechanisms driving AA by characterizing the circulating immunophenotype and faecal microbiome, and by stratifying AA to understand how identified signatures associated with heterogeneous clinical features of the condition. Flow cytometric analyses identified alterations in circulating B cells and CD4 T cells, while 16S sequencing identified changes in alpha and beta diversity in the faecal microbiome in AA. The proportions of transitional and naïve B cells were found to be elevated in AA, particularly in AA samples from individuals with >50% hair loss and those with comorbid atopy, which is commonly associated with extensive hair loss. Although significant changes in circulating CD8 T cells were not observed, we found significant changes in CD4^+^ populations. In individuals with <50% hair loss higher frequencies of CCR6^+^CD4 (“Th17”) and CCR6^+^CXCR3^+^CD4 (“Th1/17”) T cells were found. While microbial species richness was not altered, AA was associated with reduced evenness and Shannon diversity of the intestinal microbiota, again particularly in those with <50% hair loss. We have identified novel immunological and microbial signatures in individuals with alopecia areata. Surprisingly, these are associated with lower levels of hair loss, and may therefore provide a rationale for improved targeting of molecular therapeutics.

## Teaser

People with alopecia areata (AA) have increased proportions of circulating CCR6+ “Th17” and CXCR3+CCR6+ “Th1/17” CD4 T cells, which are elevated significantly more often in individuals with <50% hair loss, indicating that the early stages of the disease may be more immunologically active. Changes in circulating B cells are also observed, especially in AA patients with comorbid atopy. Changes in the abundance of faecal microbiota are associated with >50% hair loss, but the direction of causation is not yet defined.

## Graphical Abstract

**Graphical Abstract:**
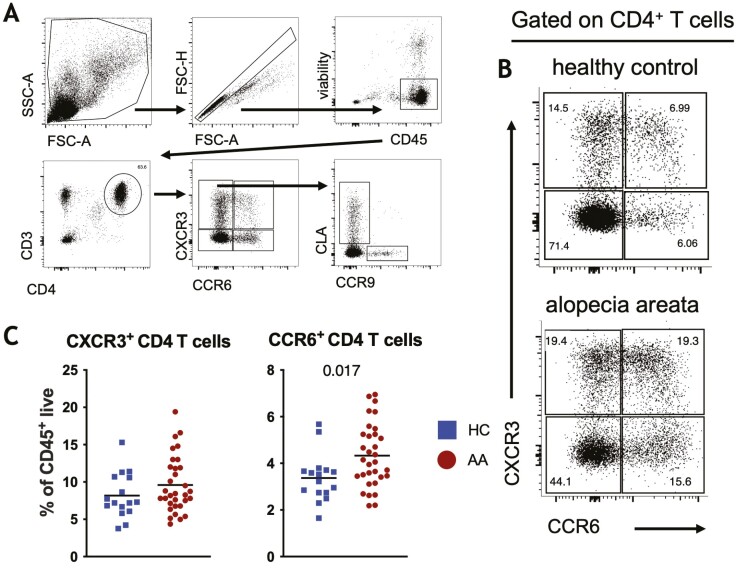

## Introduction

Alopecia areata (AA) is an immune-mediated disease that causes hair loss. Hair loss can vary from a single patch to multiple patches or may progress to affect the entire scalp and body. The condition is associated with a 2% lifetime risk [1], making it one of the most common immune-mediated diseases, and is often associated with comorbid inflammatory conditions, including atopy [2]. Current treatments, including intralesional corticosteroid injections, topical contact sensitizers, and systemic immunosuppressants, may promote hair regrowth but are often ineffective for severe diseases [3, 4]. This lack of efficacy has driven the investigation of targeted molecular therapeutics, including inhibitors of Janus kinases, which have been assessed in recent trials (clinicaltrials.gov: NCT0357074; NCT03732807). To inform the development of the next generation of therapeutics, it is important to understand the immune mechanisms underlying AA pathology.

Damage to hair follicles in AA is mediated by T-cells. Hair loss occurs as a consequence of follicular immune privilege collapse leading to activation of autoreactive NKG2D^+^CD8 T cells [5]. Mechanistic studies have focused on the pathogenic role of CD8 T cells and IFNγ, but little is known about the involvement of other lymphocytes, despite evidence indicating broad immune dysregulation. Systemic changes in immune cells are observed in AA. Specifically, levels of type 17 and type 2 cytokines, and related chemokines, are elevated compared to normal controls [6–9], and phenotyping studies report increased proportions of circulating IL-17^+^ and IL-13^+^CD4 T cells [10, 11]. In AA skin, molecules associated with distinct CD4 T cells are upregulated, and CD4 T cells are abundant in follicular infiltrates [11, 12]. Notably, CD4 T cells may be essential for the development of extensive AA, as observed using the C3H/HeJ mouse model of AA [13, 14], highlighting their potentially important helper function to CD8 T cells in AA.

In addition to CD4 T cells, autoantibodies specific for follicular epitopes are elevated in AA circulation [15]. However, current evidence does not indicate a direct pathogenic role for autoantibodies [16, 17], although the potential involvement of other B cell functions has ll mediate not been investigated. Notably, inflammatory diseases including coeliac disease [18] and type 1 diabetes (T1D) [19] have a CD8 T cell-mediated disease component, but also involve CD4 T cells and B cells. Importantly, investigating the wider contribution of the immune system to tissue pathology has been vital for understanding these diseases. Collectively, these data indicate that a more detailed investigation of CD4 T cell and B cell populations may contribute to understanding AA pathology.

At present, a lack of understanding of how the immune response may vary between people with AA is limiting the development and testing of targeted therapies. Whilst it is known that acute AA is associated with dense follicular lymphocytic infiltrate [20], we do not fully understand how this response may differ between individuals with patchy or more extensive hair loss. Furthermore, AA is often associated with comorbid inflammatory conditions but the relationship between these pathologies in relation to potentially shared biology is not understood. Atopy, for instance, is very common in people with AA [21] and is associated with poor prognosis, suggesting atopic mechanisms might exacerbate AA [22–24].

In addition, whilst the genetic factors influencing AA are well characterized [25], the impact of environmental factors is less clear. Previous studies investigating the AA microbiota identified changes in the abundance of specific bacteria [26–28]. Such changes in the intestinal microbiota are often described as potential environmental drivers of systemic inflammation [29–32], but can also occur as a consequence rather than a driver of inflammation [33].

Our aim was to investigate AA immunopathogenesis by phenotyping circulating populations of CD4 T cells, CD8 T cells, and B cells. We also sought to investigate the AA intestinal microbiome using 16S sequencing of stool samples. We hypothesized that cellular and microbiome changes will be associated with particular groups, thus individuals with AA were stratified based on their atopic status or severity of hair loss.

Our findings extend studies indicating that AA is associated with a systemic inflammatory phenotype. Consistent with published work [34] we identify an association between a B cell signature and atopic AA. In addition, we observe a novel CD4 T cell signature that is surprisingly most prominent in those with less severe hair loss, perhaps indicating that these individuals may benefit most from emerging immune-targeting therapeutic agents. This group of AA patients with <50% SALT score also has reduced microbial Shannon diversity, like that seen in other inflammatory conditions [35, 36]. Thus our data delineate changes in the systemic immune response and the intestinal microbiota that occur during the immunologically active earlier stages of hair loss in AA.

## Results

### Study participants

In total, 65 adult participants with a diagnosis of AA were recruited, alongside 38 age- and sex-matched healthy controls (HCs). AA diagnosis was made clinically at a specialist hair clinic at the Queen Elizabeth University Hospital. Severity of Alopecia Tool (SALT) scores, treatment, and atopic status of participants recruited for phenotyping and microbiome studies are described in Table 1. Atopic phenotype was defined as a positive history of one or more of the following diagnoses: atopic dermatitis, allergic asthma, and allergic rhinitis. In the phenotyping cohort, 21 AA patients were atopic, including 6 patients who were positive for all 3 conditions, 6 were positive for 2 conditions, and 9 were positive for one condition. In the microbiome cohort, 27 AA patients were atopic, including 5 patients who were positive for all 3 conditions, 7 were positive for 2 conditions, and 15 were positive for one condition. There is some overlap between the cohorts, which have identical recruitment criteria.

**Table 1:** Demographics of participants recruited for blood phenotyping and faecal microbiome analyses.

Blood phenotyping		
	Alopecia areata	Healthy controls
Total	37	20
Female/male, *n*	31/6	16/4
Mean age ± SD, years (range)	43 ± 12 (23 – 67)	39 ± 11 (23 – 57)
Mean disease duration ± SD (range), years	17 ± 12 (1 – 46)	
Low SALT (<50%)	11	
High SALT (>50%)	23	
Remission	3	
Body hair loss	31/37	
Atopic	21	
Thyroid related condition	6	
Treatment, *n*		
None	21	
IL steroid	8	
Diphencyprone	7	
Methotrexate	1	
**Faecal microbiome**		
Total	41	19
Female/male, *n*	39/2	18/1
Mean age ± SD, years (range)	45 ± 13 (18 – 68)	41 ± 14 (25 – 70)
Mean disease duration ± SD (range), years	16 ± 15 (1 – 55)	
Low SALT (<50%)	16	
High SALT (>50%) Remission	18 5	
Body hair loss	29/41	
Atopic	27	
Thyroid related condition	8	
Treatment, n		
None	22	
IL steroid	12	
Diphencyprone	5	
Methotrexate	2	

We used the antibodies described in Supplementary Table S1 to analyse circulating leukocyte populations in peripheral blood using flow cytometry. We used the markers bound by these antibodies to identify 74 discrete cell populations, including sub-populations of B cells, CD4 T cells, CD8 T cells, natural killer cells, and dendritic cells. Here we report where differences were observed in cell proportions between AA and HCs.

### Distinct B cell subpopulations are elevated in AA circulation

**Figure 1:**
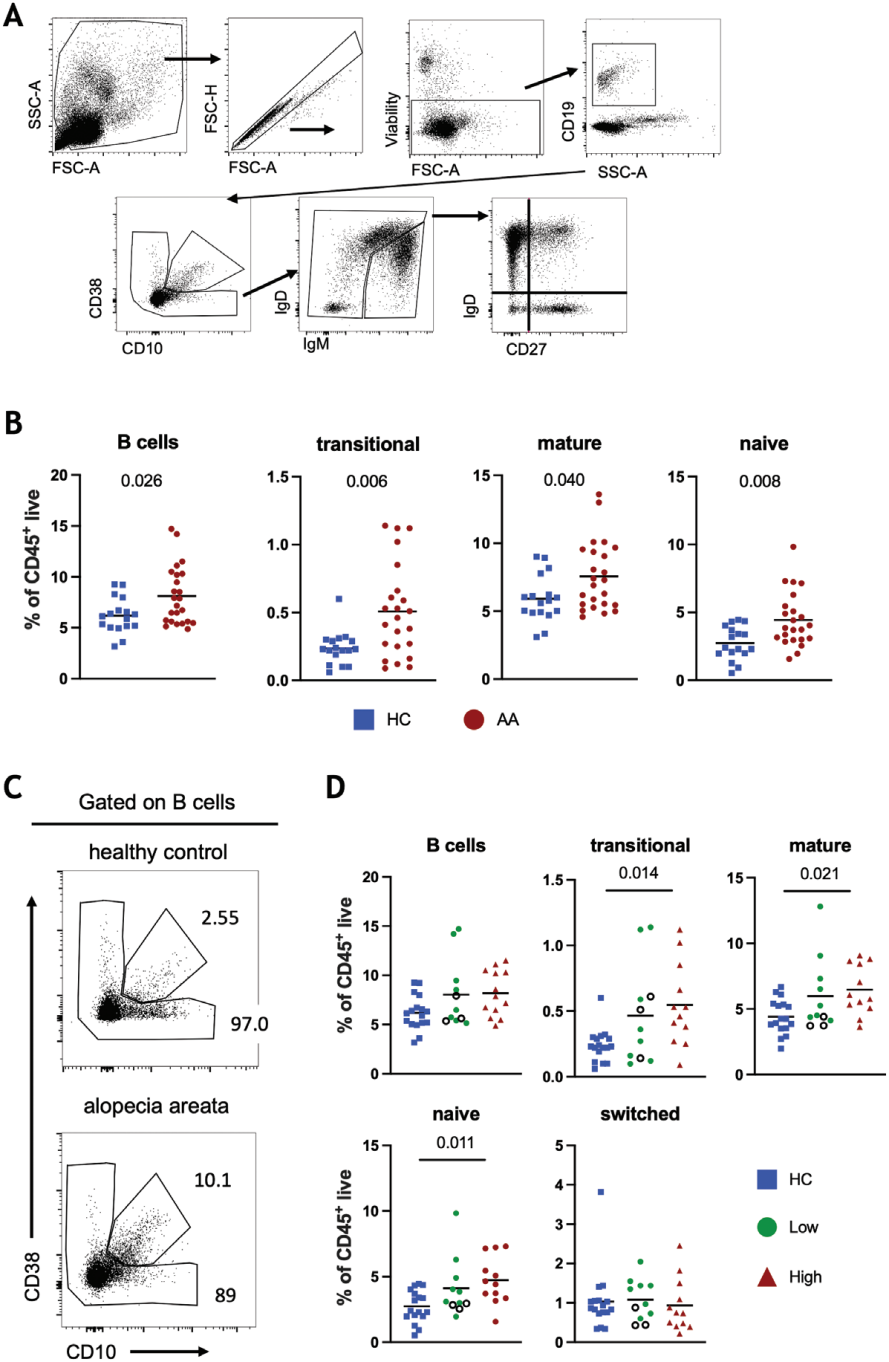
Circulating B cell profile of AA. (a) Gating strategy for identification of CD45^+^ live B cell subsets; transitional (CD38^+^CD10^+^), mature (non-transitional cells), naïve (IgD^+^CD27^−^), un-switched (IgD^+^CD27^+^), switched (IgD^−^CD27^+^) and IgD^−^CD27^-^. (b) Frequencies of total B cells, transitional, mature and naïve B cells as a proportion of CD45^+^ live cells in AA patients and HCs. (c) Proportions of total B cells, transitional, mature, naïve, and switched B cells in low AA (<50% SALT), high AA (>50% SALT), and HC cohorts. Empty circles represent patients experiencing disease remission at time of recruitment. (d) Representative plots of transitional and mature B cell frequencies as a proportion of B cells. *P < 0.05, **P < 0.01, Mann–Whitney *U*-test or Kruskal–Wallis test with Dunn’s multiple comparison.

To investigate potential associations between B cells and AA, we assessed the frequency of B cell populations in AA patients and HCs (Fig. 1a). The frequency of circulating CD19^+^B cells, transitional (CD38^+^CD10^+^), and mature B cells (all non-transitional B cells) were elevated in AA compared to HCs (Fig. 1b and c). We found no change in the frequency of class-switched (IgD^−^CD27^+^) B cells; this population includes plasma cells (IgD^−^CD27^++^). However, the proportion of naïve (IgD^+^ CD27^−^) B cells was elevated in AA compared to HCs (Fig. 1b). To determine whether these B cell changes correlated with the severity of the clinical disease the AA participants were stratified into two groups, with either low (<50% SALT) or high (>50% SALT) levels of hair loss. This stratification revealed that the proportions of transitional and naive B cells were specifically elevated in the high AA group, but not in the low AA group when compared to HCs (Fig. 1d).

**Figure 2:**
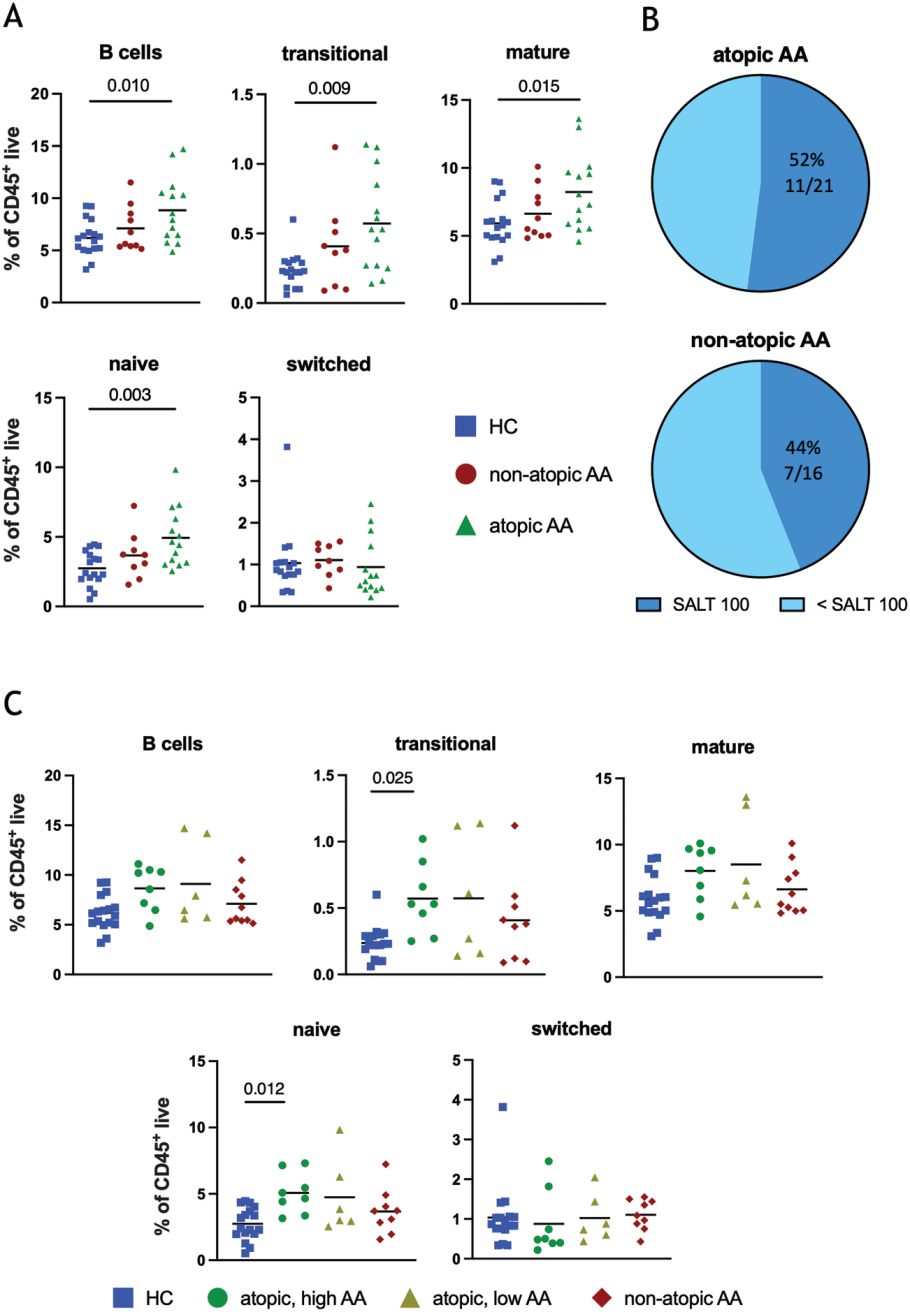
The relationship between the B cell signature and atopy. (a) Frequencies of total B cells, transitional, mature, naïve, and switched B cells as a proportion of CD45^+^ live cells in atopic AA, non-atopic AA, and HC cohorts. (b) Pie chart representation of proportions of the patient with a SALT score of 100 in the atopic AA cohort (top) and non-atopic AA cohort (bottom). (c) Frequencies of total B cells, transitional, mature, naïve, and switched B cells in atopic high or low SALT, non-atopic AA, and HC cohorts. *P < 0.05, **P < 0.01, Kruskal–Wallis test with Dunn’s multiple comparison or one-way ANOVA with Tukey’s multiple comparisons test according to normality testing.

Comorbid atopy is common in people with AA [21], and B cells contribute to atopic inflammation. Thus, we hypothesized that the observed B cell signature may be associated with atopy. Stratification of AA participants into atopic and non-atopic groups revealed that the frequency of total B cells, transitional, mature and naïve B cells are indeed increased in the atopic AA cohort compared to HCs (Fig. 2a).

These analyses indicated that a B cell signature exists in individuals with AA who are atopic, and in individuals with more severe AA (>50% SALT). An association between atopy and severe AA has previously been observed [22]. Our data are consistent with this association, indicating a somewhat higher incidence of severe AA (SALT 100) in the atopic (52%, 11/21) cohort compared to the non-atopic (44%, 7/16) cohort (Fig. 2b), although this difference was not significant. We hypothesized that the high SALT and atopic cohort represent an overlapping pathotype. To investigate this, the AA cohort was stratified according to atopic status in addition to their AA disease severity (high and low). These analyses indicated that the significantly higher proportions of transitional and naïve B cells were found specifically in the atopic, high SALT group compared to HCs (Fig. 2c).

We also investigated whether the B cell signature was related to treatment status. Despite the small sizes of some of the treatment groups, we observed an increase in the proportions of mature and naïve B cells in the IL steroid-treated AA group, and an increase in the proportion of transitional B cells in the DCP-treated AA group compared to HCs (Supplementary Fig. S1a). The frequency of total B cells was not changed in any comparison.

### Proportions of CD8 T cell populations are not altered in AA circulation

**Figure 3:**
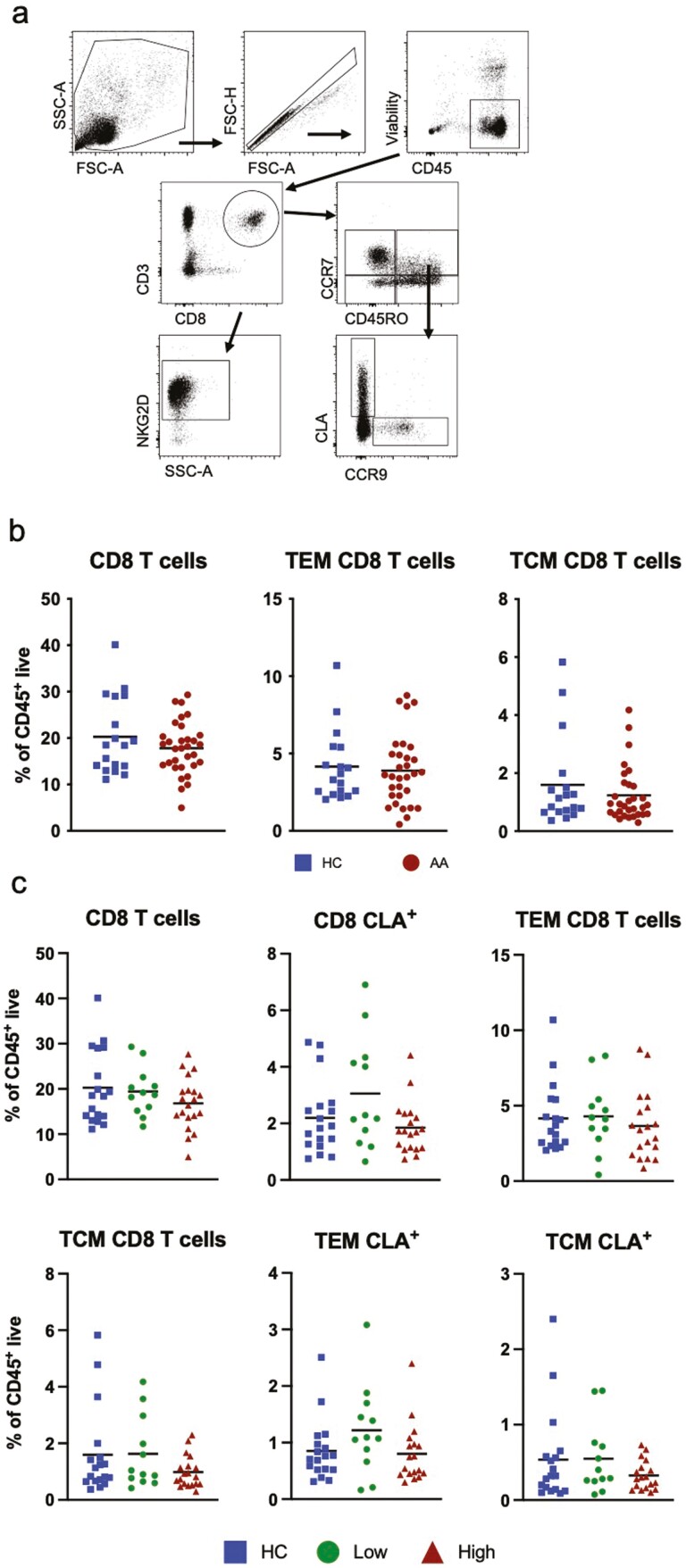
Circulating CD8 T cell profile of AA. (a) Gating strategy for identification of CD45^+^ live CD8 T cell subsets; naïve (CCR7^+^CD45RO^−^), effector memory (TEM, CCR7^-^CD45RO^+^), central memory (TCM, CCR7^+^CD45RO^+^) and terminally differentiated effector (TEMRA, CCR7^−^CD45RO^−^). CD8 T cell expression of skin (cutaneous lymphocyte antigen, CLA) and gut homing (CCR9) markers, in addition to NKG2D expression were also identified. (b) Frequencies of CD8 T cells, TEM CD8 T cells and TCM CD8 T cells in AA patients and HCs. (c) Frequencies of CD8 T cells, CLA^+^ CD8 T cells, TEM CD8 T cells, TCM CD8 T cells, CLA^+^ TEM cells and CLA^+^ TCM cells in low AA (<50% SALT), high AA (>50% SALT) and HC cohorts.

The proportions of CD8 T cell populations are reported to be altered in AA circulation [10]. We assessed circulating CD8 T cells in our cohort (Fig. 3a) but found no changes in the frequencies of total CD8 T cells, central memory (TCM, CCR7^+^CD45RO^+^), effector memory (TEM, CCR7^-^CD45RO^+^), terminally differentiated effector (TEMRA, CCR7^−^CD45RO^−^), or naïve (CCR7^+^CD45RO^−^) CD8 T cells between AA and HCs (Fig. 3b). Stratification of the AA cohort based on SALT score also revealed no significant changes (Fig. 3c). We also investigated whether there were differences in CD8 T cells between atopic and non-atopic patients but observed no changes (data not shown).

### Proportions of CD4 T cell populations are elevated in AA circulation

**Figure 4.**
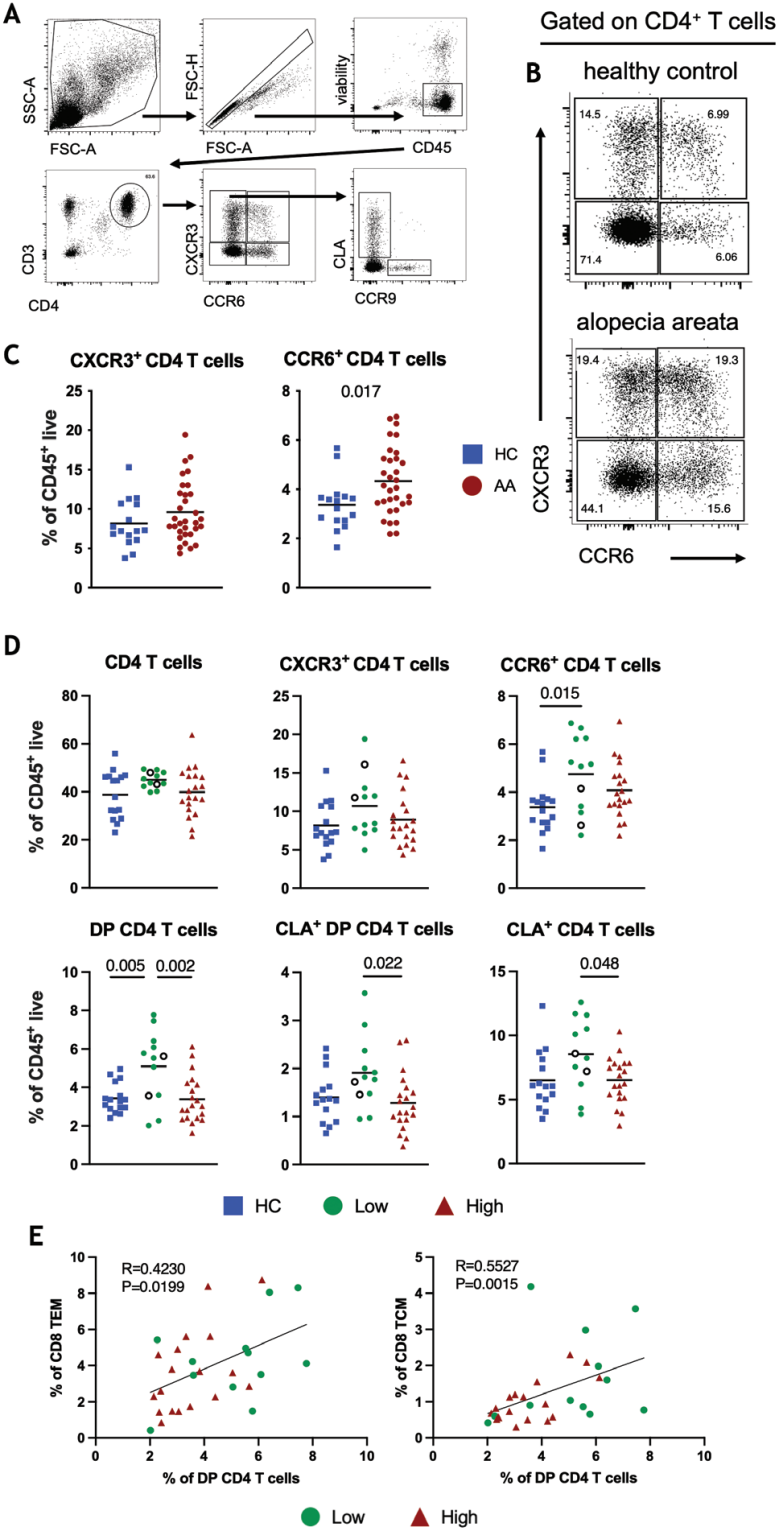
Circulating CD4 T cell profile of AA. (a) Gating strategy for identification of CD4 T cells. CXCR3 and CCR6 were used as surrogate markers to identify Th1 and Th17 cells, and CLA and CCR9 were used to identify skin and gut homing cells, respectively. (b) Representative plots of CCR6^+^, CCR6^+^CXCR3^+^, CXCR3^+^, and CCR6^−^CXCR3^−^CD4 T cells. (c) Frequencies of CCR6^+^ and CXCR3^+^ CD4 T cells. (d) Frequencies of CD4 T cell subsets in low AA (<50% SALT), high AA (>50% SALT), and HC cohorts. Empty circles represent patients experiencing disease remission. (e) Correlation of the proportion of DP (double positive, CXCR3^+^CCR6^+^) CD4 T cells with central (TCM) and effector memory (TEM) CD8 T cells. *P < 0.05, **P < 0.01, unpaired *t*-test or one-way ANOVA with Tukey’s multiple comparisons test.

We next assessed the proportions of circulating CD4^+^T cells (Fig. 4a). For this analysis, we utilized the chemokine receptors CCR6 and CXCR3 as surrogate markers for identifying Th17 [37] and Th1 cells [38], respectively. The frequency of total CD4^+^T cells (data not shown) and CXCR3^+^CD4 “Th1” T cells were not changed, however, the proportions of CCR6^+^CD4 “Th17” T cells were significantly elevated in AA compared to HCs (Fig. 4b and c). Stratification based on the SALT score revealed an increase in the proportion of CCR6^+^CD4 T cells in those with low AA (<50% SALT) compared to HCs (Fig. 4d). The proportion of CD4^+^ T cells expressing both chemokine receptors (CXCR3^+^CCR6^+^ (double positive or “DP”) CD4 T cells) was also increased in the low AA group compared to HCs, and compared to the high AA group (Fig. 4d). Skin-homing [39] CLA^+^CD4 T cells and CLA^+^DP CD4 T cells were also significantly elevated in the low AA compared to the high AA group (Fig. 4d). We also observed a significant increase in the proportion of CCR6^+^ T cells in those with body hair loss involvement compared to HCs (data not shown). We were underpowered to assess whether those without body hair loss (*n* = 6) also had changes in CCR6^+^CD4 T cells. No associations were found between CD4 T cell populations and atopy. Perhaps surprisingly we did not observe any significant differences between circulating leukocytes in patients with shorter (<5 years) or longer (≥5 years) disease duration in our cohort, but with only 3 patients in the shorter duration group this analysis was also underpowered.

We hypothesised that the CD4 T cell signature, identified significantly more often in the AA group with lower SALT scores, may indicate systemic immunological activity. Interestingly, the proportions of DP CD4 T cells positively correlated with proportions of central (*R* = 0.55, *P* < 0.01) and effector memory (*R* = 0.42, *P* = 0.02) CD8 T cells (Fig. 4e). Proportions of CCR6^+^CD4 T cells did not correlate with CD8 T cells.

Stratification based on treatment indicated a potential increase in the frequency of skin-homing CLA^+^CXCR3^+^CD4 T cells in the IL steroid group compared to HCs and the no treatment group, but the small number of available samples in the treatment sub-groups means that this analysis lacks discriminatory power. No changes were noted in any of the other CD4 T cell populations (Supplementary Fig. S1b).

### AA faecal microbiome

**Figure 5.**
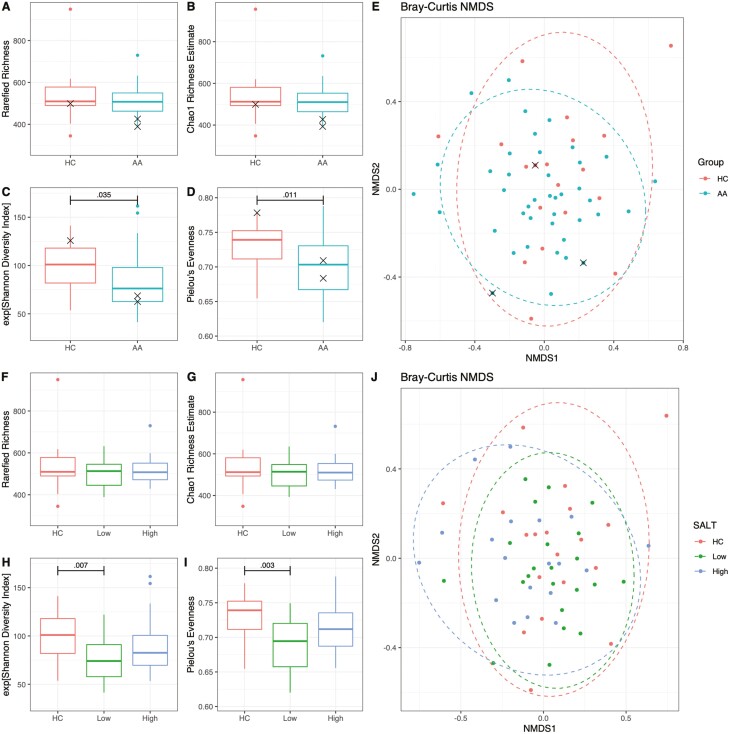
Faecal microbiome alpha and beta diversity indexes. (a, b) Rarefied and Chao1 richness, and (c, d) Shannon diversity and Pielou’s evenness estimates for AA and HC cohorts. (e) Bray–Curtis based non-metric multidimensional scaling (NMDS) plot of AA and HC samples (*R*^2^ = 0.023, adj*P* = 0.056). (f, g) Rarefied and Chao1 richness, and (h, i) Shannon diversity and Pielou’s evenness estimates for low AA, high AA, and HC cohorts. (j) Bray–Curtis based non-metric multidimensional scaling (NMDS) plot of low AA, high AA and HC samples (High AA vs HC, *R*^2^ = 0.04, adj*P* = 0.027; low AA vs. HC, *R*^2^ = 0.03, adj*P* = 0.270; low AA vs. high AA, *R*^2^ = 0.03, adj*P* = 0.270).

Alpha and beta diversity measures were used to determine changes in the AA microbiome in comparison to HCs. Alpha diversity was analysed in two ways. We first analysed sample richness, using Rarefied and Chao1 measurements, which assess the number of different types of bacteria in a sample. In addition, we analysed Shannon and Pielou’s measurements, which assess evenness and account for the abundance of each type of bacteria. Thus these evenness measurements give lower values if the community of bacteria shifts in favour of certain types of bacteria. Chao1 and Rarefied indices were not altered in AA compared to HCs, indicating no changes in microbiome richness (Fig. 5a,b). However, the evenness of the microbiome were significantly reduced in AA (Fig. 5c,d). Beta diversity measurements assess the differences between the samples in a group. The Bray–Curtis dissimilarity index was used here to compare AA and HC groups, revealing no significant difference in clustering between these groups (Fig. 5e, *R*^2^ = 0.023, adj*P* = 0.056).

Stratification of individuals with AA into groups with >50% or <50% SALT score again indicated no differences in richness between low AA, high AA, and HCs (Fig. 5f and g). However, the reductions in evenness and diversity were found, specifically in the low SALT group when compared to HCs (Fig. 5h and i).

Using these SALT-stratified groups, beta diversity was again assessed using the Bray–Curtis dissimilarity index. This analysis now indicated a difference, with the high AA group displaying higher beta diversity than the HCs. This difference explains 4% of the variance in the microbiome structure (Fig. 5j, *R*^2^ = 0.04, adj*P* = 0.027).

We next asked which bacterial taxa were contributing to this shift in beta diversity between samples from the high AA and HC groups. We identified bacteria in our samples using the amplicon sequence variant (ASV) approach, which enables more precise identification than using operational taxonomic units (OTUs); each ASV represents a bacterial population expressing a different 16S sequence. Analyses of differential abundance indicated a significant change in 18 (ASVs) between the high AA group and HCs, attributed to 13 distinct bacterial genera. No differences in the abundance of ASVs were observed between the low AA group and HCs. The enriched genera in the high AA group were from the Bacteroidetes, Firmicutes, and Tenericutes phyla, including *Alistipes* and *Bacteroides* (Supplementary Fig. S2a). In contrast, 4 genera belonging to the Firmicutes and Tenericutes phyla were reduced in high AA samples compared to HCs (Supplementary Fig. S2b). Random forest modelling indicated that 9 of these ASVs could optimally discriminate between HCs and the high SALT AA group (Supplementary Fig. S2c, OOB error rate = 21.05%, *P* < 0.001), and this model obtained an area under the ROC curve of 0.898 (Supplementary Fig. S2d).

## Discussion

We assessed patients with AA, excluding any individuals with diagnosed inflammatory or immune conditions (psoriasis, rheumatoid arthritis, ankylosing spondylitis, and inflammatory bowel diseases). Patients with atopy, common in AA, were included. Immunophenotyping indicated that AA is associated with a CD4 T cell signature, characterized by increased proportions of CCR6^+^CD4 “Th17” cells. This finding is consistent with previous studies where the proportions of IL-17^+^CD4 T cells were reported to be elevated [11]. CD4 T cells are essential for priming CD8 T cells [40], and for memory responses [41]. Thus, we hypothesized that CD4 T cells may be important during specific stages of AA pathology. Stratification based on SALT score revealed, surprisingly, that increased proportions of Th17 cells and CCR6^+^CXCR3^+^CD4^+^ “Th1/17” cells were observed significantly more often in individuals with <50% hair loss. Th1/17 cells are reported to produce both IL-17A and IFNγ [42]. In addition, the proportion of skin-homing CLA^+^CD4 T cells and CLA^+^Th1/17 CD4 T cells were increased in low compared to high AA. Overall, we observe that the increase in the Th1 and Th1/17 CD4 T cell populations is not correlated with the extent of hair loss but may indicate increased systemic immunological activity in those with lower levels of hair loss. Consistent with this hypothesis, Han et al. [11] reported increased proportions of circulating IL-17^+^CD4 T cells in those with a positive pull test, described as an ‘active’ disease.

Unexpectedly, we found no differences in the CD8 T cell compartment between AA and HCs, or in relation to disease severity or atopy diagnoses. This result is not consistent with findings from a previous phenotyping study which identified significant changes in frequencies of circulating TCM and TEM CD8 T cells, and in skin-homing TCM CD8 T cells [10]. Differences in methodology may account for the different results [10]. For instance, cells in our study were not stimulated prior to analyses, so our data reflect the phenotypes of cells directly after they are collected, rather than after they have been activated in culture. Analysis of culture-activated cells can reveal additional phenotypic differences but also introduces an additional confounding factor. Despite no change in CD8 T cells overall, our analyses did indicate that proportions of Th1/17 cells positively correlate with TEM and TCM CD8 T cell frequencies in the AA cohort. These data support the notion that elevated CD4 T cell populations are indicative of disease activity, as people with increased frequency of Th1/17 cells, also have increased proportions of CD8 T cells.

The question remains as to the contribution of Th17 or Th1/17 cells to AA pathology. Many studies have reported type 17 signatures in AA: type 17 cytokines are elevated the in blood [6, 8, 9] and factors promoting Th17 differentiation are upregulated in AA skin [43]. However, clinical blockades of IL-17A or IL-23p40, has been largely ineffective [44–46]. It may be argued, therefore, that the Th1/17 cells, especially those co-expressing CLA, could be important for maintaining CD8 T cell immunity in the skin via the production of IFNγ [47].

Our data also indicate an association between AA and altered proportions of circulating B cell populations. However, further analysis confirmed that changes in B cells were most prevalent in the atopic AA cohort. Associations have previously been identified between atopy and changes in peripheral B cell populations [48], and between atopy and severe AA [22], in addition to early onset AA, suggesting that atopy may exacerbate hair loss. Furthermore, Dupilumab (anti-IL-4Rα) has been shown to promote hair regrowth in people with comorbid atopy [49]. Indeed, we observed that severe AA (SALT 100) was more common in atopic individuals and that the changes in B cells were observed more commonly in individuals with high SALT. Thus, our data support the idea that an overlapping pathotype of atopy and high SALT exists, where atopic pathology may contribute to AA. Our study did not assess the severity of participants’ atopy; this could be addressed in future studies.

There is, as yet, no clear mechanistic connection between atopic mechanisms and CD8 T cell-mediated autoreactivity in AA. However, both the IL-13 locus [50], and molecules involved in T-B cell crosstalk including IL-2/21 [25] are associated with AA. Thus in AA, it may be hypothesized that increased proportions of transitional B cells reflect an increase in autoreactive circulating immature B cells, which could be involved in driving pathogenic T cell responses, or in modulating T cell responses, including via non-humoral mechanisms, such as presentation of self-antigen. However, it is also possible that the changes observed in circulating B cells may not directly contribute to the link between atopy and AA.

Our analyses of the microbiota indicated that the faecal microbiota richness and composition are not altered when AA samples were compared as a single group to HCs. These findings are consistent with previous studies [26, 28]. However, a recent study did report a shift in the microbiome composition in a cohort of Chinese AA patients [27]. We did, however, observe a reduction in microbial Shannon diversity and evenness in AA. However, the microbiota contributing to this change are likely to be highly heterogeneous as we did not detect any significant changes in the abundance of specific ASVs.

Stratification of AA participants into low and high SALT groups gives additional insights into microbial changes. We observe, for instance, that the reductions in evenness and diversity are found only when comparing the low SALT group with HCs. Thus the low SALT group appears to have similar stool microbiota species composition to HCs, but the microbiota in the low SALT group are distributed less evenly. This loss of evenness and diversity could reflect reduced dietary diversity in this group, or might be caused by increased levels of disturbance of the intestinal microbial ecosystem [51].

We also observed that high SALT (>50%) in AA is associated with a shift in beta diversity, compared to HCs (*R*^2^ = 0.04, adj*P*=0.027). This change is characterized by altered abundance of 18 ASVs, we found that *Alistipes*, *Bacteroides,* and *Barnesiella* were enriched in AA. Notably, a recent study also reported an increased abundance of *Alistipes* in those with severe AA (>50% SALT) [27]. The potent producers of short-chain fatty acids, *Lachnospiraceae* and *Ruminococcaceae*, were reduced in AA samples from the high AA group. As in other diseases [29, 30], the observed changes in the microbiota may not be causative in AA but may be a consequence of systemic inflammation which itself contributes to disease deterioration. In addition, differences in dietary habits between participants may contribute to changes in the gut microbiota [52, 53]. As we do not observe significant differences between the microbiota in the high and low AA groups, and because immunological and microbiological analyses were largely performed on different groups of donors, we are unable to comment on potential causal relationships between the microbiota and the immune parameters we have described.

We report here that specific subgroups of AA patients display altered CD4 T cell and B cell signatures. We suggest that AA with atopy may represent a distinct pathotype, which more commonly associated with severe hair loss. We also hypothesize that the AA patients we find with a Th1/Th17 immunophenotype, who are more likely to have a SALT score of <50%, may be more likely to respond to targeted immunotherapy. While our findings are potentially important, this study was cross-sectional and exploratory. We propose that studies now be designed that test the notion that escalation of therapeutic intervention should be considered prior to extensive hair loss. Future longitudinal studies will also be essential to investigate pathogenic mechanisms and prognostic factors in this dynamic and heterogeneous disease.

## Patients and methods

This study was exploratory in nature and sought to investigate relationships between AA and features of the immunophenotype and faecal microbiome.

### Participant recruitment

Participants were recruited to the Glasgow AA Research Clinic at the Queen Elizabeth University Hospital. Consented adults without diagnosis of a secondary autoimmune/inflammatory disease, including psoriasis, rheumatoid arthritis, ankylosing spondylitis, and inflammatory bowel diseases, were enrolled under research ethics committee approval (West of Scotland REC 1, 17/WS/0029). Age- and sex-matched healthy controls (HCs) were recruited at the University of Glasgow under ethics approved by the College of Medical, Veterinary and Life Sciences Ethics Committee (Ref: 200180145). Disease severity was determined using the severity of alopecia tool (SALT) [54] and participants were stratified into two groups: low (<50%) and high (>50%). Atopic status was confirmed by the positive history of eczema and/or asthma and/or hayfever.

### Flow cytometry

Peripheral blood was collected in lithium heparin vacutainers (BD Biosciences, US). PBMCs were extracted by density gradient centrifugation using Histopaque-1077 (Sigma, UK), stained for viability (eBioscience, US) and FC-receptor blocked (eBioscience, US). Cells were stained with antibodies (Supplementary Table S1) in Brilliant Stain Buffer (BD Biosciences, US) to generate a detailed immune-phenotype. Cells were washed, fixed (Fixation buffer, Biolegend) and acquired on a BD LSR Fortessa at the Flow Core Facility (University of Glasgow). Data were analysed using FlowJo (V10.2).

### Faecal microbiome

DNA was extracted from freeze-dried stool according to manufacturer’s protocol (MoBio Powersoil DNA kit, MoBio, USA). The V4 16s rRNA region was amplified by qPCR and amplicons were sequenced on a MiSeq instrument (Illumina) by Novogene (China) using paired end reads (2 × 250 base pairs).

### Bioinformatics

Amplicon Sequence Variants (ASVs) were generated from the raw data using the dada2 pipeline [55] (https://benjjneb.github.io/dada2/tutorial.html). Data were quality filtered (supporting appendix) and ASVs were taxonomically classified to genus level against the SILVA 132 16S reference dataset [56] using the assign taxonomy function in dada2.

### Statistical analysis

Statistical differences between the two groups were determined using an un-paired *t*-test or Mann–Whitney *U* test. Differences between three or more groups were determined using a one-way ANOVA with a Tukey’s multiple comparisons test, or a Kruskal–Wallis with a Dunn’s multiple comparisons test. The Shapiro–Wilk test was used to test for normality to inform the selection of parametric or non-parametric testing. Correlations were measured using Spearman’s rank correlation tests. Statistical methods used for the microbiome data are described in Supplementary appendix.

## Supplementary Material

uxac088_suppl_Supplementary_Figure_S1

uxac088_suppl_Supplementary_Figure_S2

uxac088_suppl_Supplementary_Tables

## Data Availability

The data underlying this article will be shared on reasonable request to the corresponding author

## Abbreviations

AA: alopecia areata
ASV: amplicon sequence variant
CCR: C-C chemokine receptor
CLA: cutaneous lymphocyte antigen
CXCR: CXC chemokine receptor
DCP: diphencyprone
HCs: healthy controls
OTUs: operational taxonomic units
SALT: severity of alopecia tool
TCM: T cells, central memory
TEM: T cells, effector memory; T1D: Type 1 diabetes: .

## Permissions to reproduce

All material presented here is original and generated by the authors.

## Clinical trial registration

This study is not a clinical trial.
