# Supplementary figures and images for: Stratification of alopecia areata reveals involvement of CD4 T cell populations and altered faecal microbiota
Source: Clin Exp Immunol. 2022 Oct 6;210(2):175–86. doi: 10.1093/cei/uxac088 (PMC9750826; doi:10.1093/cei/uxac088)

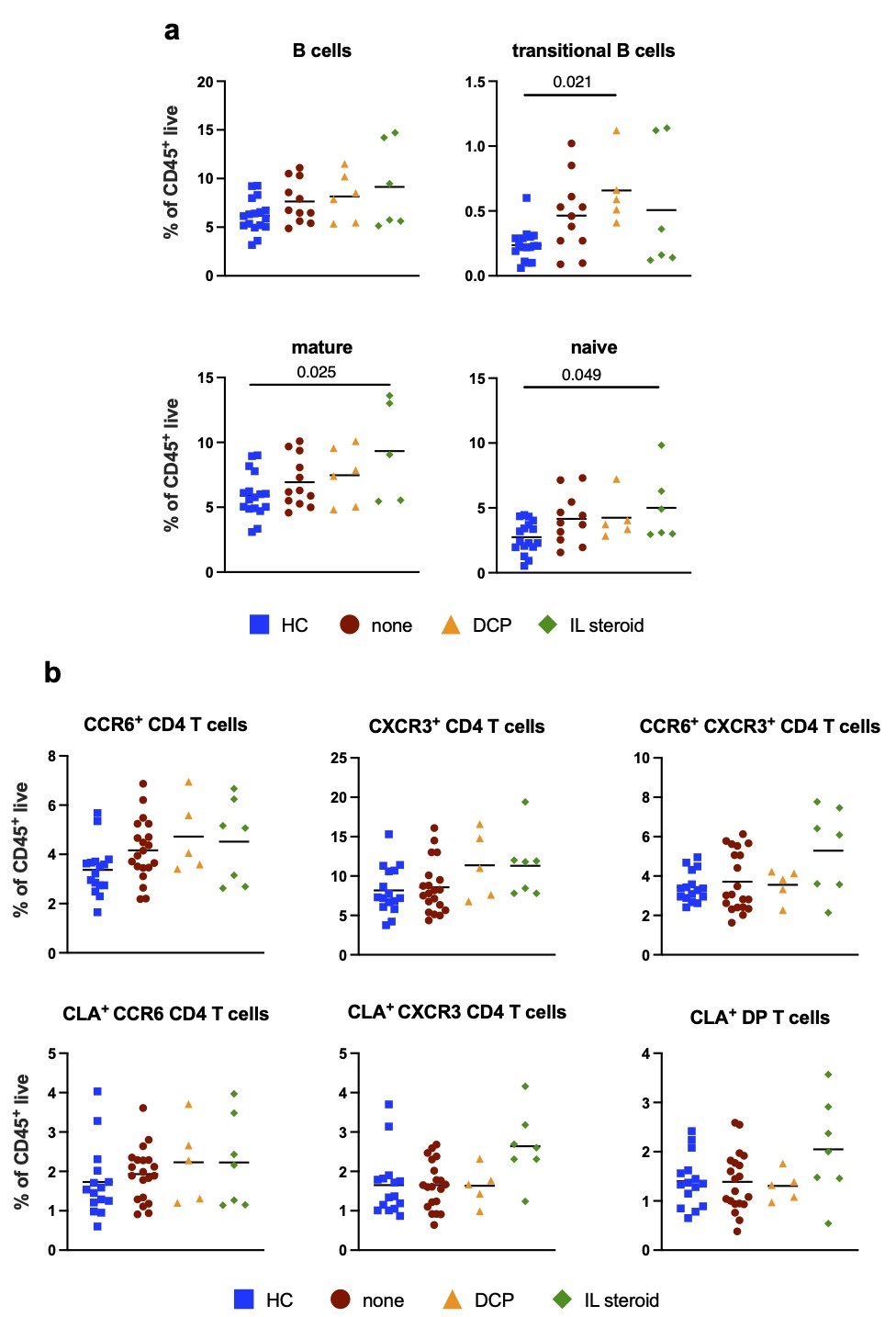

Supplement: uxac088_suppl_Supplementary_Figure_S1 [file uxac088_suppl_supplementary_figure_s1.jpeg]

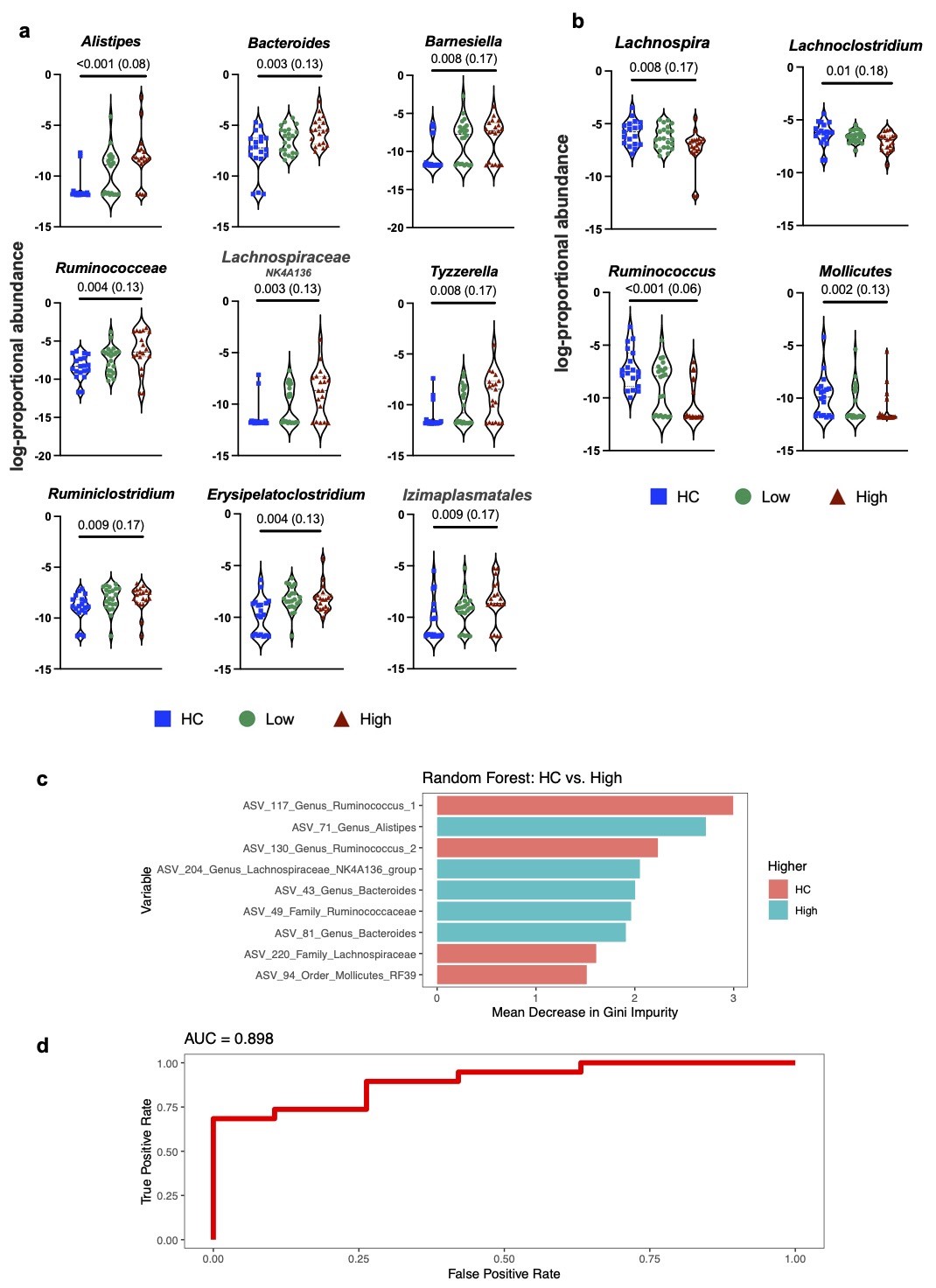

Supplement: uxac088_suppl_Supplementary_Figure_S2 [file uxac088_suppl_supplementary_figure_s2.jpeg]
