# Supplementary material for: Stratification of alopecia areata reveals involvement of CD4 T cell populations and altered faecal microbiota
Source: Clin Exp Immunol. 2022 Oct 6;210(2):175–86. doi: 10.1093/cei/uxac088 (PMC9750826; doi:10.1093/cei/uxac088)
Supplement: uxac088_suppl_Supplementary_Tables [file uxac088_suppl_supplementary_tables.docx]

**Table S1.** Antibodies used for flow cytometric analyses of PBMCs

| **Marker** | **Fluorochrome** | **Concentration** | **Clone** | **Manufacturer** |
| --- | --- | --- | --- | --- |
| CD1c | PECy7 | 1/100 | L161 | Biolegend |
| CD10 | BV605 | 1/40 | HI10a | Biolegend |
| CD11c | AF700 | 1/100 | Bu15 | Biolegend |
| CD123 | BV421 | 1/66 | 6H6 | Biolegend |
| CD127 | BV421 | 1/40 | A019D5 | Biolegend |
| CD14 | Biotin | 1/200 | HCD14 | Biolegend |
| CD141 | PE | 1/100 | IT2.2 | Biolegend |
| CD16 | FITC BUV395 | 1/200 1/200 | 3G8 3G8 | Biolegend BD Biosciences |
| CD19 | BV510 Biotin | 1/100 1/400 | HIB19 HIB19 | Biolegend Biolegend |
| CD27 | PECy7 | 1/50 | O323 | Biolegend |
| CD3 | AF700 Biotin | 1/100 1/100 | UCHT1 UCHT1 | Biolegend Biolegend |
| CD38 | AF700 | 1/40 | HB-7 | Biolegend |
| CD4 | FITC BUV395 | 1/100 1/100 | OKT4 RPA-T4 | Biolegend BD Biosciences |
| CD43 | FITC | 1/40 | MEM-59 | Biolegend |
| CD45 | BV510 | 1/100 | HI30 | Biolegend |
| CD45RA | PerCP Cy5.5 | 1/100 | HI100 | Biolegend |
| CD45RO | BV605 | 1/100 | UCHL1 | Biolegend |
| CD56 | PECy7 Biotin | 1/40 1/200 | HCD56 M-DC8 | Biolegend Miltenyi |
| CD69 | BV605 | 1/40 | FN50 | Biolegend |
| CD8 | BUV395 | 1/100 | RPA-T8 | BD Biosciences |
| CCR6 | BV605 | 1/50 | G034E3 | Biolegend |
| CCR7 | BV421 | 1/100 | G043H7 | Biolegend |
| CCR9 | APC | 1/40 | L053E8 | Biolegend |
| CXCR3 | PerCPCy5.5 | 1/50 | G025H7 | Biolegend |
| HLA-DR | BV510 | 1/100 | L243 | Biolegend |
| NKG2D | PE | 1/50 | 1D11 | Biolegend |
| CLA | FITC | 1/50 | HECA-452 | Biolegend |
| IgD | BV421 | 1/100 | IA6-2 | Biolegend |
| IgM | PerCP Cy5.5 | 1/40 | MHM-88 | Biolegend |
| Fixable viability | eFluor 780 | 1/1000 | NA | eBioscience |

**Table S2.** Summary of key findings

| Patient cohort | Immunophenotype |
| --- | --- |
| Total AA | ↑ CCR6^+^ CD4 T cells |
| Low AA: <50% SALT | ↑ CCR6^+^ CD4 T cells ↑ CCR6^+^CXCR3^+^ CD4 T cells |
| High AA: >50% SALT | Shift in the composition of the faecal microbiome |
| Atopic AA | ↑ naïve and transitional B cells |
